# Obesity and Type 2 Diabetes Mellitus Explained by the Free Energy Principle

**DOI:** 10.3389/fpsyg.2022.931701

**Published:** 2022-06-10

**Authors:** Achim Peters, Mattis Hartwig, Tobias Spiller

**Affiliations:** ^1^Medical Clinic 1, Center of Brain, Behavior and Metabolism, University of Lübeck, Lübeck, Germany; ^2^German Research Center for Artificial Intelligence (DFKI), Kaiserslautern, Germany; ^3^singularIT GmbH, Leipzig, Germany; ^4^Department of Consultation-Liaison Psychiatry and Psychosomatic Medicine, University Hospital Zurich, Zurich, Switzerland; ^5^Faculty of Medicine, University of Zurich, Zurich, Switzerland

**Keywords:** brain energy metabolism, decision making, free energy principle, obesity, selfish brain theory, stress habituation, type 2 diabetes mellitus, stress

## Abstract

According to the free energy principle, all sentient beings strive to minimize surprise or, in other words, an information-theoretical quantity called variational free energy. Consequently, psychosocial “stress” can be redefined as a state of “heightened expected free energy,” that is, a state of “expected surprise” or “uncertainty.” Individuals experiencing stress primarily attempt to reduce uncertainty, or expected free energy, with the help of what is called an uncertainty resolution program (URP). The URP consists of three subroutines: First, an arousal state is induced that increases cerebral information transmission and processing to reduce uncertainty as quickly as possible. Second, these additional computations cost the brain additional energy, which it demands from the body. Third, the program controls which stress reduction measures are learned for future use and which are not. We refer to an episode as “good” stress, when the URP has successfully reduced uncertainty. Failure of the URP to adequately reduce uncertainty results in either stress habituation or prolonged toxic stress. Stress habituation reduces uncertainty by flattening/broadening individual goal beliefs so that outcomes previously considered as untenable become acceptable. Habituated individuals experience so-called “tolerable” stress. Referring to the Selfish Brain theory and the experimental evidence supporting it, we show that habituated people, who lack stress arousals and therefore have decreased average brain energy consumption, tend to develop an obese type 2 diabetes mellitus phenotype. People, for whom habituation is not the free-energy-optimal solution, do not reduce their uncertainty by changing their goal preferences, and are left with nothing but “toxic” stress. Toxic stress leads to recurrent or persistent arousal states and thus increased average brain energy consumption, which in turn promotes the development of a lean type 2 diabetes mellitus phenotype. In conclusion, we anchor the psychosomatic concept of stress in the information-theoretical concept of uncertainty as defined by the free energy principle. In addition, we detail the neurobiological mechanisms underlying uncertainty reduction and illustrate how uncertainty can lead to psychosomatic illness.

## Introduction

In psychosomatic medicine, it is very well known that people who feel uncertain—not knowing what will happen next—are more likely to develop somatic diseases such as myocardial infarction, stroke, type 2 diabetes mellitus or obesity (Heraclides et al., 2011; Kubera et al., 2022). In 1956, the first psychosomatic workshop was held “On the mysterious leap from mind to body.” Some participants argued that “there is no separation between psyche and soma” and therefore “no leap, no mystery” (Greco, 2019). However, it was not until the beginning of the 21st century that new insights from theoretical neuroscience and internal medicine emerged that may now provide an explanation of how mental states and physical illness are related. The theoretical frameworks and experimental results underlying these explanations are presented in this article.

Stress, as redefined by Peters et al. (2017), is grounded in the concept of uncertainty. According to this novel stress definition people ask themselves on occasion, “Which of my policies should I select to safeguard my future physical, mental, and social well-being?” Those people who are *uncertain* in answering this question experience stress. Uncertainty is used here as ‘entropy’ in terms of information theory (Shannon, 1948) and means, in the context of stress, that even when people choose their most promising policy, they expect surprises, i.e., they remain uncertain about what will happen next (Hartwig et al., 2022).

The question of how the brain deals with uncertainty in general is at the center of modern theoretical neuroscience. The psychiatrist and physicist Karl Friston of the University College London has developed the so-called free energy principle according to which all sentient beings strive to reduce uncertainty or, in other words, to minimize an information-theoretical quantity he calls free energy (Friston et al., 2006; Friston, 2010). This variational free energy is measured in bits and corresponds to the information that an agent could still need to make better predictions about things in the world. So a high free energy is something unfavorable, indicating that the agent has a too complex model of reality and/or makes inaccurate predictions.

So far, the free energy principle has been successfully applied to explain various brain processes such as perception, action, attention, learning, decision making, exploration, and social cooperation (Hohwy, 2013; Schwartenbeck et al., 2013; Clark, 2016; Hartwig and Peters, 2021). Here we apply the free energy principle to stress, it being a state where all available policies of the agent display a heightened expected free energy (Hartwig et al., 2022).

The term “policy” has a special meaning in the free energy principle, specifically in the process called active inference. In active inference, beliefs about the hidden states of the world optimize model evidence, allowing to find the ‘best’ model (one that makes accurate predictions but is not too complex). In contrast to classical formulations, active inference makes a distinction between “action” as a physical state of the real world and‚ “policies”, which are beliefs about (future) actions—the latter are what constitute a sense of agency (Friston et al., 2013). The essence of active inference, namely the brain’s predictive capacity, becomes particularly evident when the agent has to make decisions under uncertainty or stress.

There are three outcomes of dealing with stress, i.e., with high expected free energy: good stress, tolerable stress and toxic stress, where the uncertainty can be reduced completely, partially or not at all (McEwen and Gianaros, 2011). Good stress is when a so-called uncertainty resolution program (URP) successfully ends the uncertainty phase so that it becomes a completed episode. Tolerable stress occurs in people who habituated to stress. Toxic stress occurs in people for whom stress habituation was not a free-energy-optimal solution and who therefore did not habituate (Hartwig et al., 2022). Compared to people who experience good stress, people with tolerable or toxic stress can develop prolonged changes in cerebral energy metabolism.

The characteristic of the brain to prioritize its own energy metabolism is described in the Selfish Brain theory (Peters et al., 2004). The Selfish Brain theory is based on the postulate that the brain actively demands energy from the body when needed, rather than just being passively supplied from the blood. Such prioritization of cerebral energy metabolism has been shown, at least in mammals, to be an inherent metabolic feature that ensures the maintenance of cerebral function, energy homeostasis and mass on short and long time scales (Sprengell et al., 2021a,b,c). Accordingly, in times of uncertainty, the brain demands additional thermodynamic energy from the body.

Cerebral energy metabolism changes in divergent directions during tolerable versus toxic stress. The cerebral energy need is decreased after stress habituation and increased during toxic stress. After stress habituation, people typically tend to develop the corpulent-but-waisted phenotype and, under toxic stress, the lean-but-narrow-waisted phenotype, the latter being associated with a high risk of cardio- and cerebrovascular complications. Both phenotypes have a high risk for type 2 diabetes mellitus (Peters and McEwen, 2015; Peters et al., 2017; Hartwig et al., 2022).

This article highlights biological mechanisms that may explain how the mental state of feeling uncertain is related to the development of different body shape phenotypes (lean, obese, narrow or wide waisted) and somatic diseases (such as type 2 diabetes mellitus, myocardial infarction, and stroke).

## Good Stress

Agents’ beliefs about their preferred goal states play a key role in understanding stress. In this paper, the term belief is understood in the Bayesian sense as a prior probability distribution over states. Goal beliefs denote the “states that the agent believes they should hold.” Typical goal states are, for example, physical integrity or a certain social status. For human agents, the goal state landscape is of course very complex. It has been shown that goal priors (beliefs) are encoded in the ventromedial prefrontal cortex (vmPFC; Bechara et al., 2000; Roesch and Olson, 2004; Barron et al., 2015), specifically as Gaussian probability distributions, where not only the mean but also the variance of the distribution is encoded (Lebreton et al., 2015). These goal or prior preferences provide a point of reference for goal directed behavior—but can also be updated, as will be shown below.

The beliefs about the “states that can be attained” (attainable states) are represented in brain regions such as the pre-supplementary motor area (pre-SMA; Rushworth et al., 2004; Nguyen et al., 2014). For example, an attainable state for an athlete is to jump 2.00 m in the upcoming competition. Athletes’ beliefs about attainable states indicate the probabilities with which the athlete believes they can attain certain jump heights. According to the free energy principle the prediction of attainable states is based on a so-called generative model (Friston et al., 2006). Past experiences—such as early life adversity or failed attachment to parents—strongly influence which future events are anticipated when choosing a particular policy. In a long-lasting iterative process, the amygdala- and hippocampus-dependent emotional and declarative memories shape the generative model, which allows the prediction of the attainable states. In this regard, no person is free from such biographical biases toward the prediction of new events.

In the anterior cingulate cortex (ACC), the risk of each available policy is assessed, allowing to deal with uncertainty in decision making (Paulus et al., 2002; Feinstein et al., 2006; Behrens et al., 2007; Sarinopoulos et al., 2010; Karlsson et al., 2012; Liljeholm et al., 2013). The policy risk assessment is based on the comparison between the predicted attainable states and the agent’s goal states (Friston et al., 2013; Peters et al., 2017). If there is a large divergence between the attainable states and the goal states (given a particular policy is considered), the policy in question is classified as high risk for not attaining the goal.

If all of the policies available to the agent are high risk, the ACC starts the URP. The URP’s primary function is to reduce uncertainty. To this end, the ACC activates the amygdala, which is at the top of a hierarchically organized stress system, involving top-down activation of brainstem nuclei, sympathetic nervous system (SNS), hypothalamic–pituitary–adrenal (HPA) axis, and bottom-up feedback of cortisol to all levels (Peters et al., 2007; Weidenfeld and Ovadia, 2017).

The URP uses information to identify a policy from the available repertoire that appears promising after all, or even to creatively find a new policy that offers a way to reduce uncertainty. Once the URP is initiated, the agent infers a stress state based on the interoceptive inputs described next. URP activation is accompanied by numerous changes in the internal milieu of the human organism (increased vigilance, faster heartbeat, sweating, and change in gastrointestinal peristalsis). The agent experiences themselves wide awake, with strong palpitations and a queasy feeling in the stomach, and interprets these changes, along with perceived external circumstances, as most consistent with feeling uncertain (Barrett and Simmons, 2015; Barrett, 2017). Although URP activation leads to an emotionally unpleasant state, it is of vital importance because it can safeguard survival by resolving uncertainty. People who succeed in resolving uncertainty by the URP experience this episode as good stress, resulting in a boost of self-esteem and a sense of mastery (Kirschbaum et al., 1995; Pruessner and Hellhammer, 1999; Pruessner et al., 2005).

The URP has three subroutines: enhancement of cerebral information processing, procurement of the necessary energy, and learning of successful measures. The first subroutine increases cerebral information transmission to reduce uncertainty. It thus increases the probability of quickly finding a promising policy. It was Claude Shannon who first showed that it requires information to reduce uncertainty (i.e., entropy; Shannon, 1948). Information facilitates the selection of states or policies under consideration, allowing the virtual reality model to make more accurate predictions, thereby increasing the likelihood of finding a successful policy.

**Figure 1:**
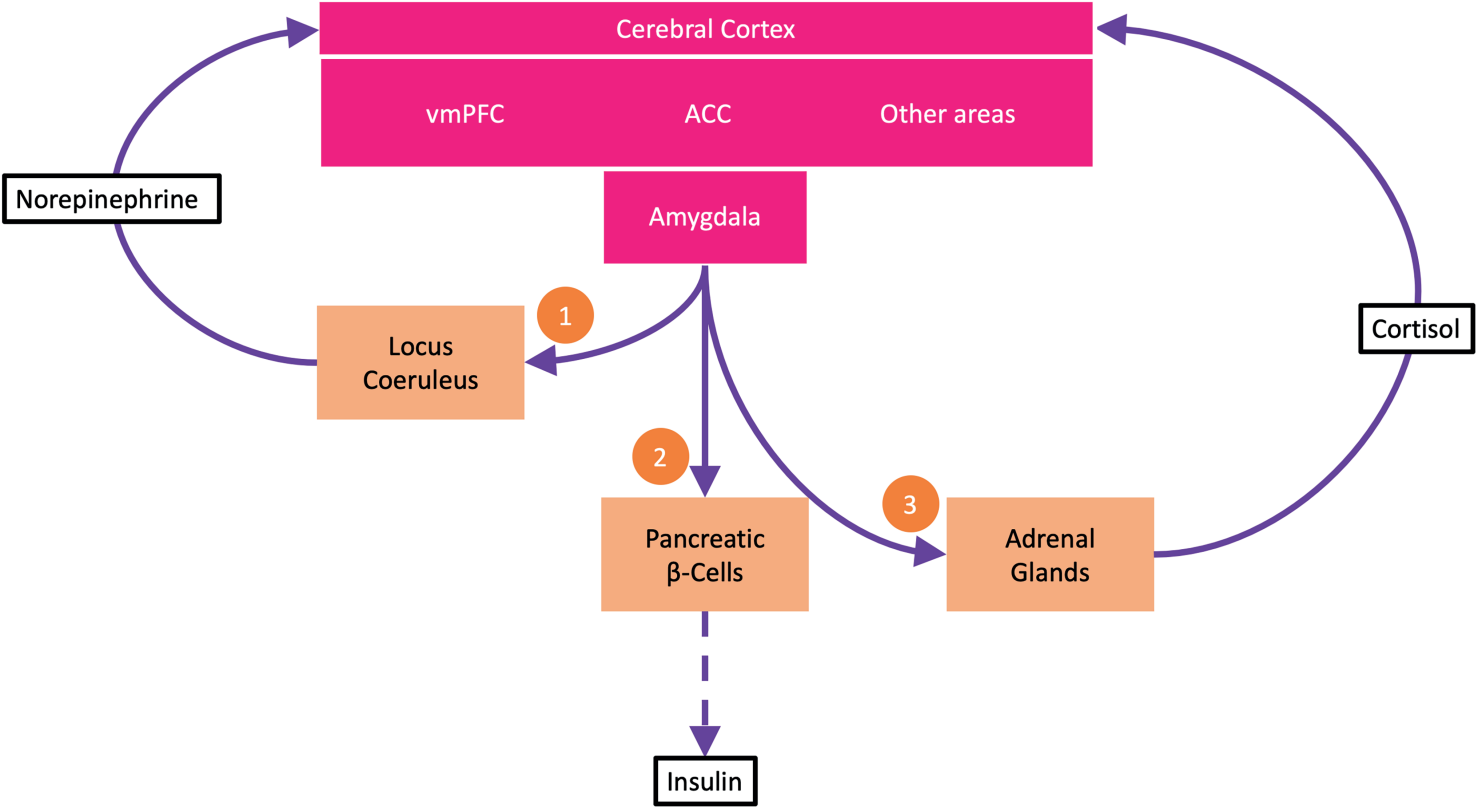
The uncertainty resolution program (URP). The ventromedial prefrontal cortex (vmPFC) encodes beliefs about goal states. Under uncertainty, i.e., when all available policies carry a high risk of not attaining the goal states, the anterior cingulate cortex (ACC) activates the URP. The URP involves three subroutines: (1) The amygdala *via* descending pathways activates neurons of the locus coeruleus, which *via* ascending projections to the cerebral cortex release norepinephrine at cortical synapses. Norepinephrine boosts cortical information processing, making information available to reduce uncertainty. (2) The amygdala demands energy from the body to support increased cerebral information processing. *Via* sympathetic efferents, the amygdala strongly suppresses the pancreatic β-cells so that insulin secretion is reduced (dashed arrow). This process is called cerebral insulin suppression. At low insulin concentrations, less glucose is available to the muscle and fat tissue (which take up glucose in an insulin-*dependent* manner) than to the brain (which takes up glucose in an insulin-*independent* manner). (3) The amygdala *via* adrenocorticotropin activates the adrenal glands to release cortisol, which feeds back to the cerebral cortex and helps decide which uncertainty resolution measures to learn for the future and which not to.

In this first subroutine, the amygdala activates locus coeruleus (LC) neurons in the brainstem (Figure 1). Tonic activation of these neurons produces an arousal state with increased vigilance. The arousal state results from the noradrenergic LC neurons sending multiple projections throughout the brain, particularly to the cerebral cortex, where they release norepinephrine at cortical synapses (Berridge and Waterhouse, 2003; Aston-Jones and Cohen, 2005). The neuromodulatory influence of norepinephrine accelerates information transmission at these synapses, allowing more information to be transmitted per time (bits per second). In this way, more information is immediately available, reducing uncertainty about what to do next—making it possible to find the most promising policy as quickly as possible.

The second subroutine procures the energy needed for speeding up cerebral information processing. For without energy, no information can be obtained (Tribus and Mcirvine, 1971; Toyabe et al., 2010; Berut et al., 2012). Indeed, experimentally induced stress arousals (i.e., states with high expected variational free energy) have been shown to be highly thermodynamic-energy costly; even a mild mental laboratory stressor results in a 12% increase in global cerebral metabolic rate of glucose (Madsen et al., 1995). In this second subroutine for energy procurement, the amygdala stimulates the SNS and the HPA-axis. In this way, the amygdala activates so-called brain-pull mechanisms with which the brain can actively demand energy from the body (Peters and Langemann, 2009). While the Selfish Brain theory postulates that the brain actively demands energy from the body when needed (Peters et al., 2004), the rival theories, i.e., the glucolipostatic theory and its modern variants, reject such an active energy demand by the brain (Kennedy, 1953; Mayer, 1953; Chaput and Tremblay, 2009; Schwartz et al., 2017). In fact, three systematic reviews have shown that the Selfish Brain theory was able to predict and coherently explain all the data examined, which covered caloric restriction, cerebral artery occlusion, and type 1 diabetes mellitus (Sprengell et al., 2021a,b,c). In contrast, the predictions of the glucolipostatic theory and its modern variants were violated. This systematic theory test showed that the postulate of an actively energy-demanding brain is probably true (Sprengell et al., 2021a,b,c).

Three of the most important brain-pull mechanisms are cerebral insulin suppression, brain-induced ketogenesis, and brain-induced heart rate acceleration. Cerebral insulin suppression involves activation of SNS and HPA-axis, both of which strongly suppress insulin secretion from pancreatic β-cells (Woods and Porte, 1974; Billaudel and Sutter, 1982; Ahren, 2000; Hitze et al., 2010). Decreased insulin release limits insulin-dependent storage of glucose in muscle and adipose tissue. Instead, the circulating glucose is available to the brain, which requires virtually no insulin for glucose uptake (Hom et al., 1984; Hasselbalch et al., 1999; Seaquist et al., 2001). In short, insulin-independent glucose-transporter-1 (GLUT-1) uptake safeguards basal energy supply of vital organs, like brain and immune cells (Deng et al., 2014), while insulin-dependent glucose-transporter-4 (GLUT-4) allows the storage of surplus energy in muscle and fat cells (Shepherd and Kahn, 1999). Put simply, enhanced cerebral insulin suppression provides the brain with extra energy when it needs it.

Brain-induced ketogenesis involves SNS stimulation of visceral adipose tissue to release free fatty acids, which travel *via* the portal vein to the liver, where they are converted to ketone bodies. Finally, ketone bodies are provided to the brain as alternative substrates when needed (e.g., during psychosocial stress or caloric restriction; Kubera et al., 2014).

Brain-induced heart rate acceleration also involves activation of the SNS. Amygdala activity is associated with heart rate variability in healthy subjects, showing a link between the processing of emotional stimuli and the corresponding adaptation of the autonomic system (Yang et al., 2007). The accelerated heart rate increases cardiac output and thus makes more energy per time available to the brain (Jones et al., 2011). Thus, brain-induced heart rate acceleration also procures the brain with energy-on-demand.

The third subroutine, learning of uncertainty-resolving measures, involves activation of the HPA-axis and cortisol feedback effects *via* brain glucocorticoid receptors. In hippocampus and amygdala, cortisol binds with high affinity to brain mineralocorticoid receptors and with low affinity to brain glucocorticoid receptors (De Kloet et al., 1998). A high cortisol concentration indicates that the URP has not yet been effective. In this case, the low-affinity brain glucocorticoid receptors are fully occupied, promoting processes such as synaptic long-term depression, thereby opposing the consolidation of declarative memory and the learning of measures (Pavlides et al., 1995; Peters et al., 2018). This makes sense, because the measures that have just been used have not been able to resolve the uncertainty, after all. In contrast, low cortisol concentrations after a stressful episode indicate that the uncertainty could be resolved and thus, under the influence of high-affinity brain mineralocorticoid receptors, consolidation of measures is favored, for example, by means of synaptic long-term potentiation (Maggio and Segal, 2007, 2009). In this way, successful measures for eliminating uncertainty are learned for use in similar future situations. Overall, the third subroutine of the URP is decisive for which measures or strategies will continue to be used in the future and which will not.

In sum, an effective URP searches for and finds a best and promising policy, provides the brain with the energy needed for the search by drawing on the body stores, and learns the selected policy, if it proves successful, for future use (Peters et al., 2017). But if the URP fails, the brain still has a last option up its sleeve to reduce uncertainty.

## Tolerable Stress

If our goal beliefs cannot be met, then as a last resort, the goal itself may be changed. It has been shown that changing goal priors can indeed reduce uncertainty (Hartwig et al., 2022). However, changing goal priors comes at a significant cost, as we have to sacrifice our deepest preferences. There is a growing body of evidence that what we call stress habituation is based on this kind of goal-belief change.

Flattening/broadening the probability distribution that reflects our goal beliefs, reduces the risk or, in other words, the expected variational free energy of the available policies (Hartwig et al., 2022). Here’s how that can be understood: In the situation where all policies fail to meet the preferred states (as indicated by the goal prior), the uncertainty for the agent is still high. Flattening the goal prior distributes the preferences (probability mass of the goal prior) over more states. Thus, there is a tendency that flattening the goal prior increases the preference of the states attainable by the available policies, in other words, the attainable states appear more acceptable to the agent. The overall shape of the goal prior is often considered as a Gaussian distribution but that is not mandatory for the concept of stress habituation (Hartwig et al., 2022). If habituation leads to policies being assessed as low risk, the uncertainty of the agent decreases. Acceptable states do not require any change or any action. Nor is there any longer a need to initiate the URP. Therefore, a flattening/broadening of goal priors leads to a lack of stress arousals and hormonal responses.

The omission of arousals and hormonal responses is exactly what characterizes stress habituation (Peters and McEwen, 2015). Stress habitation is defined as a repetition-induced response attenuation—neuroendocrine, cardiovascular, neuroenergetic and emotional—when agents are exposed to the same (homotypic) stressor. Importantly, after habituation to a homotypic stressor, the ability to provide a hormonal response to other different stressors (heterotypic stressors) is preserved (Hill et al., 2010).

In the classic habituation experiment, Kirschbaum et al. (1995) exposed probands to a Trier Social Stress Test (TSST) five times in succession. They found that two-thirds of the probands showed a steep cortisol response on the first TSST, but had virtually no cortisol response on the second to fifth repetitions. These subjects were the ones who habituated to stress. The remaining third of the probands showed no such response attenuation, and they still showed a potent cortisol response even on the fifth occasion. These subjects did not habituate. Kirschbaum’s findings indicate that stress habituation produces discrete outcomes, i.e., all-or-nothing responses. Interestingly, those who habituated (compared to those who did not) considered themselves more attractive, had higher self-esteem, and were less likely to be depressed (Kirschbaum et al., 1995). Obviously, people have a different propensity to habituate. Whether or not stress habituation occurs depends on several factors, for example, the peak cortisol levels during the stress event, genetically determined sensitivity of glucocorticoid receptors or endocannabinoid receptors (Fride et al., 2005; Malcher-Lopes et al., 2008; Hill et al., 2011; McKlveen et al., 2013). Accordingly, habituators show a high propensity and non-habituators a low propensity to habituate. We have shown recently, that habituation itself is a free-energy-optimizing process (Hartwig et al., 2022). Since the propensity to habituate varies among people, for some the habituation is the free-energy-optimal solution, for others the non-habituation.

The processes of goal preference encoding and stress habituation are colocalized in the brain. Experimental findings showed that where the goal priors are encoded, namely in the vmPFC and orbitofrontal cortex (Bechara et al., 2000; Gottfried et al., 2003; Roesch and Olson, 2004; Barron et al., 2015), the process of stress habituation is also localized; to be more precise, stress habituation is located in a subdomain of the vmPFC, that is, in the mPFC (Weinberg et al., 2010). Because the vmPFC has also been shown to encode the precision (inverse variance) of goal priors (Lebreton et al., 2015), it is plausible that habituation by reducing the goal prior’s precision leads to the absence of the cortisol responses upon re-exposure to the homotypic stressor.

The variational free energy of information theory has a pendant in physics, the Helmholtz thermodynamic free energy. The two have practically the same mathematical derivation. The variational free energy consists of an accuracy term and a complexity term.

Complexity costs arise when an update of prior beliefs leads to a new posterior belief. These complexity costs are closely related to the thermodynamic costs, i.e., the energy consumption of the brain (Sengupta et al., 2013). Sengupta and colleagues were able to show that if the information-theoretic variational free energy has a minimum (the agent has learned a great deal about the world), then the Helmholtz thermodynamic free energy has a minimum too (the brain operates in its most economical mode; Sengupta et al., 2013). Further theoretical work supports the tight entanglement of variational and thermodynamic free energy (Kiefer, 2020; Parr et al., 2020). Simply put, the better the predictions of the agent’s virtual reality model are (as accurate as possible, but not too complex), the fewer calories their brain burns.

People who have habituated are prone to exhibit sub-average brain energy consumption (Peters et al., 2017). This is because they experience fewer or no brain-energy-costly stress arousals. In contrast, for people who live a good and vibrant life, stressful episodes that occur from time to time are quite normal. They are a manifestation of the activated URP, which helps to immediately resolve (sometimes unavoidable) uncertainty. Thus, compared to people who live a vibrant and good life, people who have habituated to stress hardly show any arousal states, which leads to a sub-average energy need of the brain.

To understand what a reduction in brain energy consumption means for energy metabolism in the body, it is helpful to look at the brain’s energy supply chain. The cerebral supply chain is a formalized representation of the Selfish Brain theory (Peters and Langemann, 2009). Accordingly, the brain is viewed as an end consumer of a supply chain, in which energy flows from the environment through the body to the brain. In logistics and economics, the mathematical laws of supply chains have been known for 70 years (Slack et al., 2004). These principles are applied here to human energy metabolism. The key feature of this cerebral supply chain is the brain-pull. The brain-pull is the function that enables the brain to actively demand energy from the body when needed. The theory test mentioned above used three systematic reviews to examine all possible bottlenecks within the cerebral supply chain, one bottleneck affecting inflow to the whole organism (caloric restriction), one to the brain (cerebral artery occlusion), and one to muscle and adipose tissue (type 1 diabetes mellitus); the test result was that a brain-pull function was indispensable for making accurate predictions (Sprengell et al., 2021a,b,c). As examples of neuroendocrine mechanisms that take over brain-pull function, we have already mentioned cerebral insulin suppression and brain-induced heart rate acceleration. Body-pull is known from conventional metabolic models and refers to the function that enables the body to demand energy from the environment when needed. Body-pull is commonly called ingestive behavior.

When the brain needs less energy, a supply chain build-up can occur (Peters and McEwen, 2015). If customers do not buy, the shelves stay full. In terms of human energy metabolism, this means that if the brain consumes less energy, a corresponding reduction in brain-pull function will occur. When cerebral insulin suppression is attenuated, β-cells secrete more insulin at a given blood glucose level, blood insulin levels rise inappropriately, and more glucose is stored in muscle and adipose tissue (because peripheral glucose uptake is insulin-dependent). In this way, hyperinsulinemia promotes the accumulation of energy in body stores, and when this occurs over years, obesity is likely to result (Odeleye et al., 1997).

When the body stores are already heavily filled, further uptake of glucose into the stores becomes increasingly difficult, for two reasons: insulin resistance and relative insulin deficiency. Insulin resistance develops when energy accumulation in muscle and fat cells increases the concentration of O-linked β-N-acetylglucosamine (O-GlcNAc), a marker of total cell energy content (Ong et al., 2018). O-GlcNAc inhibits insulin-dependent glucose uptake *via* GLUT-4 (Vosseller et al., 2002).

Relative insulin deficiency occurs when increased O-GlcNAc concentrations in muscle and adipose cells stimulate leptin release from these tissues (Wang et al., 1998; Einstein et al., 2008); increasing leptin concentrations activate the SNS *via* a hypothalamic circuit, thereby leading to SNS-mediated insulin suppression (Mizuno et al., 1998). SNS-mediated insulin suppression, in turn, leads to a relative decrease in blood insulin concentration. The combination of insulin resistance and relative insulin deficiency makes it difficult for glucose to be stored further in muscle and fat tissue. Of note, both insulin resistance and relative insulin deficiency are considered here to have a common cause, a central nervous event, namely a reduction in brain energy consumption induced by stress habituation. In this way, habituation can cause energy to accumulate in the body, first in adipose tissue and then as glucose in the bloodstream. Just as shelves stay full when customers do not buy, energy builds up in the body when the brain needs less energy. If the blood glucose concentration rises above a certain level, type 2 diabetes mellitus is diagnosed.

In summary, stress habituation—by eliminating stress arousals and thus lowering brain energy consumption—can lead to a build-up in the cerebral supply chain: energy accumulating in the body stores in the form of triglycerides promotes the development of obesity; energy accumulating in the bloodstream in the form of glucose promotes the development of type 2 diabetes mellitus. Thus, people who habituated are prone to develop an obese type 2 diabetes mellitus phenotype.

Acceptable conditions, even if they are hostile to life, do not necessarily prompt action to change the situation, as noted earlier. For habituated people, their lack of need-for-change (resulting from their acceptance of conditions) may contribute to their being stuck in precarious circumstances such as poverty or low social status. It should be noted that stress habituation is a learning process that identifies situations that can be survived without taking action. Stress habituation is a subconscious free-energy-optimizing process. Therefore, people who had to give up their goal preferences due to stress habituation cannot be held responsible for their obese phenotype or their precarious social condition. Obesity and type 2 diabetes are also known to be associated with poverty and low social status (Mohammed et al., 2019). Experimental evidence has shown that the opportunity to move out of poverty areas reduces participants’ uncertainty and the prevalence of obesity and type 2 diabetes (Ludwig et al., 2011, 2012). On this background, it is conceivable that uncertainty and stress lead to stress habituation (in those who have a propensity to habituate), with habituation being a common cause of staying put in adverse social circumstances on the one hand and developing the obese type 2 diabetes phenotype on the other.

## Toxic Stress

If uncertainty cannot be resolved by either the URP or habituation, toxic stress arises (Peters et al., 2017). When uncertainty persists and expected free energy remains high, the person enters a state of recurrent or permanent stress arousal, even at night, and this can manifest as insomnia. The agent under toxic stress searches for a promising policy, cannot find it, and gets frustrated. Unlike a person who habituates and consequently becomes passive, the person suffering from toxic stress remains in search and still has the chance to find a primary solution, i.e., a successful measure. You could also say non-habituators bet everything on one card—win or perish. This distinguishes them from habituators, who compromise between reducing uncertainty and accepting precarious conditions.

People who suffer from toxic stress are prone to exhibit an above-average brain energy consumption. In toxic stress, the recurrent and permanent brain energy-consuming arousal states during the day and the lack of brain energy-saving deep sleep at night contribute to the overall increase in total brain energy consumption. This is because normally cerebral energy consumption is lowered by 40% during deep sleep (Maquet et al., 1990; Boyle et al., 1994). Thus, in a prolonged state of high expected free energy, the brain incurs high thermodynamic energy costs.

A recurrent or permanent increase in brain energy need has far-reaching consequences for energy metabolism in the body. Increased cerebral energy need corresponds with increased activity of brain-pull mechanisms, especially the aforementioned cerebral insulin suppression, brain-induced ketogenesis, and brain-induced heart rate acceleration (Peters and McEwen, 2015).

When cerebral insulin suppression is enhanced, β-cells secrete less insulin at a given blood glucose level, blood insulin levels become inappropriately low, and less glucose is stored in muscle and adipose tissue (since the peripheral glucose uptake requires insulin). Such limitation of peripheral glucose uptake makes sense because the glucose circulating in the blood is now available to the brain with its high energy requirements. When this preferential allocation of energy to the brain rather than to the body continues for longer periods of time, weight loss occurs, resulting in a more or less pronounced lean phenotype.

During toxic stress, when glucose uptake into peripheral energy stores is severely limited, a build-up of energy in the supply chain may occur, but this is different from that which occurs after habituation. Under toxic stress, energy accumulates in the form of glucose in the bloodstream because glucose can hardly enter the peripheral stores. Once the blood glucose concentration exceeds a certain level, type 2 diabetes mellitus is diagnosed. That psychosocial stress is a cause of type 2 diabetes mellitus is supported by numerous randomized controlled trials: anti-stress interventions such as cognitive behavioral therapy lower blood glucose concentrations or glycosylated hemoglobin (Chapman et al., 2015; Xu et al., 2021). Taken together, people experiencing toxic stress tend to develop a lean type 2 diabetes mellitus phenotype.

When toxic stress lasts longer, it leads to the accumulation of visceral fat, a hallmark of chronic stress (Bjorntorp, 2001). Under the influence of chronic glucocorticoid excess, abdominal fat cells divide and become able to take up more glucose (Kuo et al., 2007; Peters and McEwen, 2015). In this way, energy is stored in the visceral fat tissue in the form of triglycerides. Whenever the brain needs extra energy, which is often the case under toxic stress, it can draw on this energy store in the abdomen. This cerebral energy demand occurs through brain-induced ketogenesis, which can be summarized as follows: the SNS stimulates visceral adipose tissue to release free fatty acids, which are converted in the liver into ketones, serving as alternative fuels for the brain. In light of these findings, visceral adipose tissue can be viewed as an outsourced extracerebral energy depot that the brain can draw upon when it needs energy. Epel et al. demonstrated in a habituation experiment that in women who underwent repeated TSSTs, there was a stronger cortisol response in those with increased abdominal fat than in those without. This was particularly the case in lean women who did not habituate to repeated stress (Epel et al., 2000). In all, chronic toxic stress can lead to the development of the lean-but-wide-waisted type 2 diabetes phenotype.

When the brain-induced heart rate acceleration persists under toxic stress, it can have detrimental effects on the cardiovascular system (Peters et al., 2017). Blood circulating at high velocity through the vascular system can cause turbulences, especially at the arterial bifurcations (Malek et al., 1999). At these predilection sites, turbulences can trigger mechanisms that can lead to plaque formation, atherosclerosis, and, in the worst case, myocardial infarction or stroke (Stone et al., 2012). There is strong evidence that people with the lean-but-wide-waisted phenotype have the greatest cardiovascular risk and the obese-but-narrow-waisted phenotype have the least (Berentzen et al., 2010; Petursson et al., 2011; Cameron et al., 2012; Hamer and Stamatakis, 2012; Ortega et al., 2013; Krakauer and Krakauer, 2014; Hu et al., 2018; Berglund et al., 2019; Kim et al., 2019; Kubera et al., 2022). That psychosocial stress is a cause of myocardial infarction is supported by two randomized controlled trials: an anti-stress program, i.e., cognitive behavioral therapy, could lower the incidence of cardiovascular events and mortality (Orth-Gomer et al., 2009; Gulliksson et al., 2011).

Non-habituated and habituated individuals show the following differences.

While stressed non-habituated individuals are more likely to exhibit the wide waisted phenotype (accumulation of visceral fat), habituated individuals do not display glucocorticoid excess, which would promote visceral fat accumulation, and therefore more likely retain the narrow waisted phenotype (Peters and McEwen, 2015). In addition, stressed non-habituated individuals lose subcutaneous fat and tend to become lean, whereas habituated individuals accumulate subcutaneous fat and tend to gain weight. In short, non-habituators are prone to develop the lean-but-wide-waisted phenotype, habituators the obese-but-narrow-waisted phenotype. Because habituation is specific and only ever relates to a particular homotypic stressor, someone may habituate to one stressor at work but not to another homotypic stressor at home. Such a case would result in a mixed phenotype, obese with a wide waist (i.e., body mass and waist circumference increased). That is, interaction between a complex environment and a non-homogeneous population (consisting of habituators and non-habituators) can result in a wide range of phenotypes: at one end of the spectrum, the lean-but-wide-waisted phenotype with high cardiovascular risk; at the other end, the obese-but-narrow-waisted phenotype with low cardiovascular risk; at the two ends, the lean and obese phenotypes, respectively, both with an equally high risk for type 2 diabetes mellitus (Feller et al., 2010).

## Conclusion

In this article, we understand stress as a state of uncertainty or, in technical terms, a state with heightened expected variational free energy. We outlined how the brain has two options to reduce uncertainty or expected free energy. First, the brain can use an URP (stress arousal, SNS and HPA-axis response, control of learning) to immediately reduce uncertainty and learn successful measures. If the URP is successful, the agent experiences good stress. When the URP fails, stress habituation is an alternative for some—but not all—people to reduce uncertainty. We show evidence that habituation minimizes free energy by a flattening/broadening of goal preferences. Reducing the precision of goal preferences (i.e., goal priors) means that the agent accepts adverse (previously untenable) states, such as lower social status or poverty. People who habituated experience tolerable stress. We detailed how habituation decreases the average brain energy consumption, what in the long run may lead to the development of the obese type 2 diabetes mellitus phenotype. In some stressed people, no habituation occurs because for them habituation itself is not the free-energy optimal solution; in these cases, toxic stress results. Toxically stressed people have been searching in vain for a way out of their uncertainty, but at least they still have a chance to find that way out, e.g., through the help of others. Toxic stress increases the average brain energy consumption, what in the long run can lead to the development of lean-but-wide-waisted type 2 diabetes mellitus. An increased brain energy consumption, by overloading the cardiovascular system, can also lead to the development of myocardial infarction and stroke.

By linking the free energy of information theory with that of thermodynamics, brain energy need comes into focus. Cerebral energy metabolism can be understood as bridging the gap between uncertainty and metabolic disease. In the conventional models of obesity and type 2 diabetes mellitus, cerebral energy need was not even considered as a factor. However, systematic theory testing revealed that the postulate of a brain that actively demands energy according to its needs is probably true (Sprengell et al., 2021a,b,c). Future research should clarify the extent to which the newly identified factor of erebral energy need contributes (completely, partially or not at all) to the observed increases in body mass and blood glucose concentrations.

In summary, using the principle of free energy, we have shown three different ways in which the brain can deal with uncertainty and what consequences this can have for the body, such as obesity or type 2 diabetes mellitus.

## Author Contributions

All authors listed have made a substantial, direct, and intellectual contribution to the work and approved it for publication.

## Conflict of Interest

MH is employed at singularIT GmbH, Leipzig, Germany.

The remaining authors declare that the research was conducted in the absence of any commercial or financial relationships that could be construed as a potential conflict of interest.

## Publisher’s Note

All claims expressed in this article are solely those of the authors and do not necessarily represent those of their affiliated organizations, or those of the publisher, the editors and the reviewers. Any product that may be evaluated in this article, or claim that may be made by its manufacturer, is not guaranteed or endorsed by the publisher.
